# *In Vitro* Analysis of Integrated Global High-Resolution DNA Methylation Profiling with Genomic Imbalance and Gene Expression in Osteosarcoma

**DOI:** 10.1371/journal.pone.0002834

**Published:** 2008-07-30

**Authors:** Bekim Sadikovic, Maisa Yoshimoto, Khaldoun Al-Romaih, Georges Maire, Maria Zielenska, Jeremy A. Squire

**Affiliations:** 1 Department of Pediatric Laboratory Medicine, The Hospital for Sick Children, Toronto, Ontario, Canada; 2 Genetics and Genome Biology Program, The Hospital for Sick Children, Toronto, Ontario, Canada; 3 Division of Cellular and Molecular Biology, Department of Research, Ontario Cancer Institute (OCI), University Health Network (UHN), Toronto, Ontario, Canada; 4 Department of Pathology and Molecular Medicine, Richardson Labs, Queen's University, Kingston, Ontario, Canada; Wellcome Trust Sanger Institute, United Kingdom

## Abstract

Genetic and epigenetic changes contribute to deregulation of gene expression and development of human cancer. Changes in DNA methylation are key epigenetic factors regulating gene expression and genomic stability. Recent progress in microarray technologies resulted in developments of high resolution platforms for profiling of genetic, epigenetic and gene expression changes. OS is a pediatric bone tumor with characteristically high level of numerical and structural chromosomal changes. Furthermore, little is known about DNA methylation changes in OS. Our objective was to develop an integrative approach for analysis of high-resolution epigenomic, genomic, and gene expression profiles in order to identify functional epi/genomic differences between OS cell lines and normal human osteoblasts. A combination of Affymetrix Promoter Tilling Arrays for DNA methylation, Agilent array-CGH platform for genomic imbalance and Affymetrix Gene 1.0 platform for gene expression analysis was used. As a result, an integrative high-resolution approach for interrogation of genome-wide tumour-specific changes in DNA methylation was developed. This approach was used to provide the first genomic DNA methylation maps, and to identify and validate genes with aberrant DNA methylation in OS cell lines. This first integrative analysis of global cancer-related changes in DNA methylation, genomic imbalance, and gene expression has provided comprehensive evidence of the cumulative roles of epigenetic and genetic mechanisms in deregulation of gene expression networks.

## Introduction

The hallmark of cancer is the deregulation of gene expression profiles and disruption of molecular networks [1]. Mutation and genomic instability provide tumours with sufficient diversity, so that cells with adaptive and proliferative selective advantage can evolve in a Darwinian manner. However, it has become evident that epigenetic factors, particularly heritable changes in DNA methylation, may confer additional and more diverse advantage to tumours including deregulation of gene expression and destabilization of chromatin. While there is some understanding of how such genetic and epigenetic changes may influence the gene expression, and thereby tumour evolution, it is less clear how these mechanisms influence each other, and how cumulative changes could co-evolve and influence gene expression during tumourigenesis.

Many human diseases have been linked to aberrant DNA methylation or mutations in the DNA methylation pathways [2], but the most compelling evidence of DNA methylation disorders and human pathogenesis is evident in cancer. Malignant cells can show major disruptions in DNA methylation profiles, which manifest as aberrant hypermethylation and hypomethylation of gene promoters, as well as global genomic hypomethylation [3]. To date, many genes with aberrant promoter hypermethylation have been identified in tumours, including cell cycle regulators, DNA repair genes, genes associated with apoptosis, hormonal regulation, detoxification, metastasis, angiogenesis, and many others [4], [5].

Another type of cancer-related defect of DNA methylation is genomic hypomethylation [6]. It is common in both solid tumours such as prostate cancer [7], hepatocellular cancer [8], cervical cancer [9], as well as in hematologic cancers such as B-cell chronic lymphocytic leukemia [10]. Decreased levels of global DNA methylation can be indicative of tumour progression in many types of malignancies including breast, cervical and brain cancers [6]. Aberrant hypomethylation has been hypothesized to contribute to cancer progression by activating oncogenes such as *h-RAS*, *r-RAS*, *c-MYC*, and *c-FOS* [11]–[13], by retrotransposon activation [14]–[16] or by increasing chromosome instability as in ICF syndrome [17].

Osteosarcoma (OS) is the most common primary bone malignancy, and is characterized by complex chromosomal abnormalities that vary widely from cell to cell. These tumours exhibit high degree of aneuploidy, gene amplification, and multiple unbalanced chromosomal rearrangements. A combined approach of molecular cytogenetic techniques [comparative genomic hybridization (CGH), spectral karyotyping (SKY), multicolour banding (mBAND), or array-CGH (a-CGH)] together with the classical G-banded cytogenetic analysis of OS tumours have described complex karyotypes with multiple numerical and structural chromosomal aberrations. Collectively these studies highlight the highly unstable nature of the OS genome [18]–[29].

Although genetic changes in OS have been extensively researched, our understanding of epigenetics in this tumour is very limited. Only a handful of studies reported changes in DNA methylation in OS, and no genome-wide DNA methylation analysis has been published. Changes in promoter methylation of osteocalcin gene have been suggested to play a role in osteoblast differentiation and carcinogenesis in human and rat [30]–[32]. Aberrant DNA methylation of the imprinted *IGF2* and *H19* loci has also been observed and suggested to play a role in OS tumourigenesis [33]. The *RASSF1* gene was shown to be frequently hypermethylated and as re-expressed by decitabine, in a panel of pediatric tumours including OS [34]. We have recently reported the first genome-wide study of gene expression in OS cell line, by assessing the changes in expression of U2OS cells in response to decitabine exposure [35]. In this study it was shown that decitabine-induced DNA demethylation in a number of pro-apoptotic genes including *GADD45A* resulted in apoptosis of OS cell lines U2OS and MG63, and mouse xenografts of OS cultures, drawing attention to the therapeutic potential of modulating DNA methylation [36].

Recent developments in microarray technologies have revolutionarized the way in which DNA methylation is studied. In addition to using methylation-sensitive restriction enzymes, methylated genomic DNA has been successfully enriched using immunoprecipitation with a 5-methylcytosine antibody (Me-DIP) and used for hybridization to genomic microarray platforms [37], [38]. These advancements have allowed for very precise mapping of genome-wide epigenetic profiles. For example, the first genome wide characterization of epi-toxicogenomic changes of histone acetylation profiles at a thirty five nucleotide resolution using Affymetrix Promoter Tiling arrays was reported [39].

The functional relevance of epigenetic changes in cancer aetiology is only beginning to be deciphered. Since acquired changes in gene expression may be influenced by both genetic and epigenetic factors in humans, the objective of this study was to develop an approach for integration of global cancer-specific epigenomic and genomic profiles with gene expression, and study these changes in two well-characterized OS cell lines. Our previous published studies of MG63 and U2OS have demonstrated that both these cell lines have the typical complex genomic structure that characterizes patient derived osteosarcomas. Because we have a detailed knowledge and have published extensively on the genomic biology of both these particular cell lines we are uniquely positioned to assess the validity and technological utility of the integrated whole genome analytical approach using this in vitro system. Furthermore, our goal was to develop a high-resolution approach of detection of genome-wide and gene-specific changes in DNA methylation.

Using integrative epigenetic, genome imbalance, and gene expression analyses we provided first genomic DNA methylation maps, and identified and validated genes with aberrant DNA methylation in osteosarcoma cell lines. These analyses provide evidence of the cumulative roles of epigenetic and genetic mechanisms in deregulation of gene expression networks in OS cell lines.

## Results

**Figure 1:**
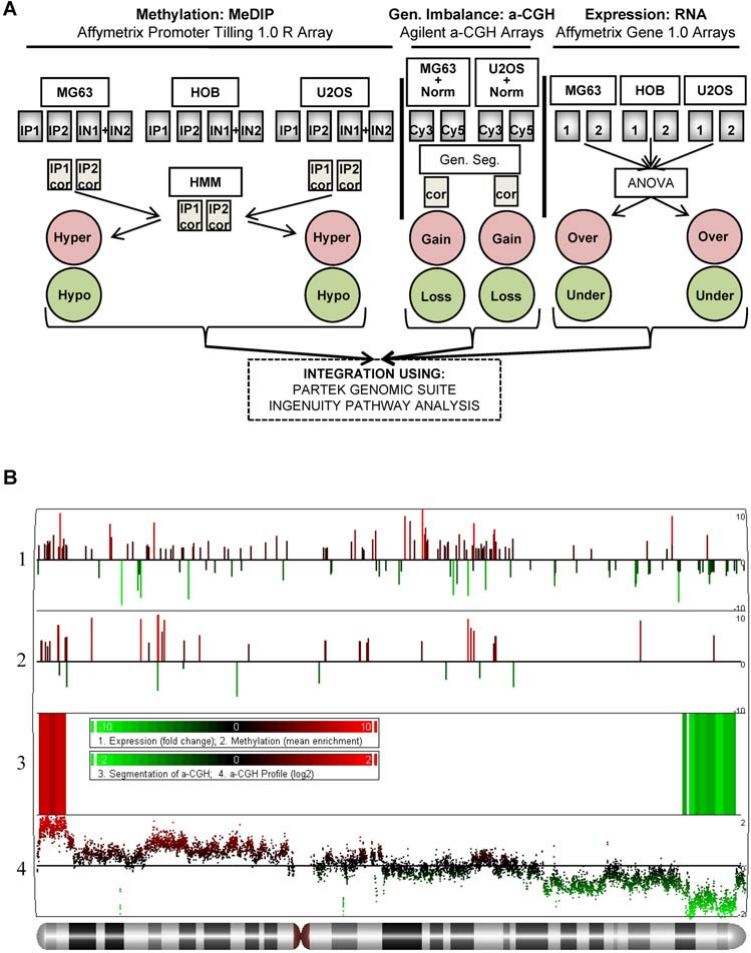
Integrative epigenetic, genetic, and expression profiling. (A) Schematic workflow of microarray data analysis and integration. Individual microarrays in replicates (grey boxes), are imported, background corrected, and significantly enriched or depleted regions are detected and assigned to specific genes for DNA methylation, genomic imbalance, and gene expression. All data were analyzed, and integrated using Partek Genomic Suite (PGS) software, and network analysis was performed using Ingenuity Pathway Analysis (IPA). IN – input DNA, IP – immunoprecipitated DNA Cy3 and Cy5 – replicate experiment dye flip for a-CGH labeling, 1 and 2 – individual expression replicates, cor. – background normalized/corrected arrays, HMM – Hidden Markov Model algorithm, gen. seg. – genomic segmentation algorithm, ANOVA – analysis of variance algorithm. (B) Chromosome view of epigenetic, genetic, and expression changes at chromosome 7 in U2OS cells. A PGS generated visualization of the significant regions of genes with significant changes in expression (lane 1 profile), DNA methylation (lane 2 profile), genomic segmentation algorithm results (lane 3 heat map), and the corresponding a-CGH profile (lane 4 profile). The genomic segmentation scale and a-CGH profile values are in log_2_, while DNA methylation and gene expression y-axis scale represents average fold change to osteoblast levels, and size of each bar is proportional to the fold change (green – decrease, red – increase).

To assess genome wide changes in DNA methylation related to OS that may play a role in deregulation of gene expression, as well as to delineate the potential genomic imbalance contributions, an integrative and functional approach for the analysis of DNA methylation, genomic imbalance and gene expression was created, using two OS cell lines, U2OS and MG63, and normal human osteoblasts. Figure 1A shows a schematic outline of the analysis with detailed protocol description in the methods. The representative chromosome view of one of the chromosomes (chromosome 7) with differentially methylated and differentially expressed genes, as well as regions of significant genomic imbalance for one of the cell lines (U2OS) is shown in Figure 1B.

### High resolution DNA methylation analysis

Affymetrix Promoter 1.0 Tilling Array platform covers 10–12.5 kb regions (2.5 Kb 3′ and 7.5–10 Kb) of 25,500 human gene promoters, with an average tilling resolution of 35 nucleotides. This platform was previously used for profiling of genomic histone acetylation in a breast cancer model of environmental exposures [39]. We utilized this platform in combination with the methylated DNA immunoprecipitation (Me-DIP) to develop a comprehensive approach for detection of hypo- and hypermethylation changes at high resolution, and used it to detect such changes in human OS cells in relation to the normal osteoblasts.

In order to assess the quality and reproducibility of our Me-DIP-chip procedure the hierarchical clustering of the raw data was performed using Partek Genomic Suite (PGS) software before and after the background normalization (Figure S1A). This analysis clearly showed the differential clustering of the Metlyl-C immunoprecipitated (IP) versus the input (IN) samples across all 12 arrays, as well as differential clustering of OS (U2OS and MG63) cells in comparison to osteoblasts both before and after normalization. The good agreement between the replicate array “heatmaps” within each cell type illustrates the high level of reproducibility of the Me-DIP-chip protocol. Similar clustering results were reproduced with the Principal Component Analysis of the raw data (Figure S1B).

We next proceeded to detect the significantly differentially enriched regions in each of the cancer cell lines, with osteoblast levels as baseline. In order to exploit the high resolution of this platform the Hidden Markov Model algorithm was used with specific parameters (see methods) to allow for detection of relatively short regions at minimum of 10 probes and approximately 350 nucleotides with strongly enriched/depleted regions (s-HMM), as well as longer and overall less enriched/depleted genomic regions with minimum 15 probes and approximately 500 nucleotides (m-HMM), and 40 probes or approximately 1400 nucleotides (l-HMM). This analysis resulted in identification of 828 s-HMM, 763 m-HMM, and 582 l-HMM in U2OS cells (Table S1), and 641 s-HMM, 529 m-HMM, and 407 l-HMM regions in MG63 cells (Table S2). The relative enrichment mean across the probes, representing the fold enrichment in relation to osteoblasts in a specific HMM region, ranged from +35.8 to −24.7 in U2OS, and from +20.1 to −21.6 in MG63 cells. As expected, a large proportion of the three types of HMM-detected regions overlapped same genomic locations. For example, promoter region of one of the gene, *LHX9*, was detected as significantly enriched in both U2OS and MG63 cells by both s-HMM and l-HMM algorithm, while shorter, but more enriched s-HMM region represented a sub-section of the larger, but less enriched l-HMM region (Figure S2).

These regions were then annotated to specific gene loci using the Affymetrix HuGene-1_0-st-v1.na24.hg18.transcript.csv library. We have identified 831 enriched/hypermethylated and 397 depleted/hypomethylated gene promoters in U2OS (Table S3) and 440 enriched/hypermethylated and 359 depleted/hypomethylated gene promoters in MG63 cells (Table S4).

### Gene-specific validation of Me-DIP-chip

**Figure 2:**
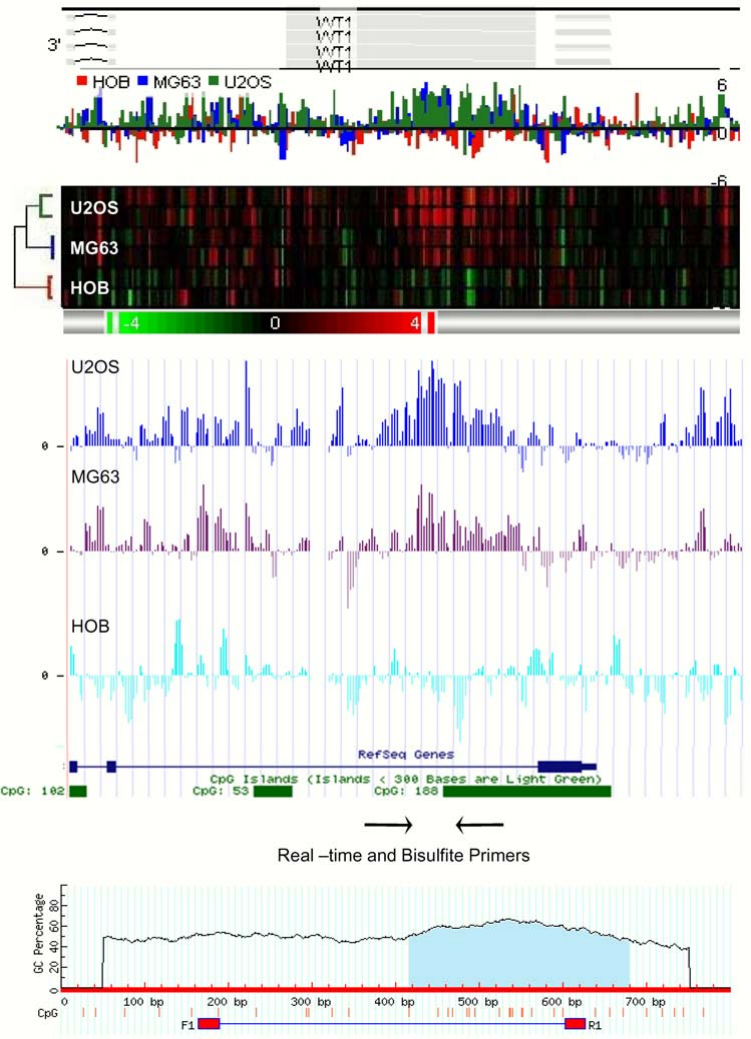
Visualization and validation of Me-DIP-chip detected regions. Top panel is the PGS-generated region view of the enriched/hypermethylated genomic region (grey box) in the *WT1* gene promoter, featuring the colour-coded profile of the signal from each cell type (in log_2_), and the corresponding heat-map of the replicate array experiments bellow (in log_2_). Note the reproducibility of the signal, as well as the dendrogram of the hierarchical clustering for the region to the left of the heat maps. Middle panel shows the PGS-generated .wig file of this region imported into UCSC Genome Browser mapped to the *WT1* gene promoter, and the corresponding CpG island, allowing identification of genomic features of interest at 35 nucleotide resolution, and design of the primers for the validation experiments including the real-time PCR, and bisulfite EpiTYPER quantitation of DNA methylation. Bottom panel represents the MethPrimer-generated view of the region and the corresponding CpG dinucleotides (red bars) whose methylation levels are quantitated using EpiTYPER.

The Me-DIP-chip protocol utilized the enrichment of methylated DNA with the methyl-C-specific antibody, as well as the processing of the IP and IN DNA including the whole-genome amplification, DNA fragmentation, labeling, hybridization, scanning and software analysis. In order to validate the technical aspects of analysis gene-specific real-time PCR quantitation of the IP enriched DNA was performed. In addition, the quantitative analysis of the cytosine methylation in a number of genes in both IP and IN fractions of U2OS, MG63, and normal osteoblasts was carried out. Figure 2 is a representative schematic of the design of such validation experiments in *WT1*, which is one of the genes detected as enriched in both U2OS and MG63 relative to normal osteoblasts.

**Figure 3:**
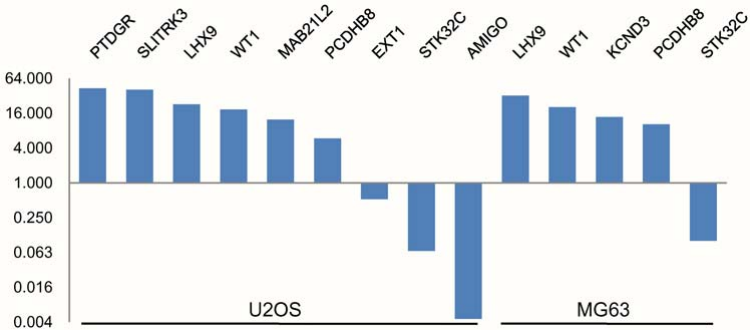
Gene-specific real-time PCR validation of the Me-DIP-chip data. Me-DIP-chip detected genes in Table 1 were subject to real-time PCR quantitative analysis of enrichment. The y-axis represents fold enrichment values generated by calculating the ration of U2OS or MG63 IP (Ct)/IN(Ct) over osteoblast IP(Ct)/IN(Ct). Each real-time PCR reaction was performed in triplicate and average values were used for enrichment calculation.

Several genes were detected as either enriched or depleted in either U2OS and/or MG63 cells relative to the osteoblast levels (Table 1) and used for the real-time PCR validation, using the original, non-amplified IP and IN DNA that was used in the Me-DIP-chip experiment. Figure 3 shows relative enrichment (IP/IN) of U2OS or MG63 versus normal osteoblasts. All fourteen gene-specific real-time PCR experiments confirmed the microarray findings.

**Table 1:** Me-DIP-chip detected genes for real-time PCR and EpiTYPER validation.

Chromosome	Gene Name	Gene Symbol	Status
*U2OS*			
1	LIM homeobox 9	LHX9	Enriched
5	protocadherin beta 8	PCDHB8	Enriched
11	Wilms tumor 1	WT1	Enriched
3	SLIT and NTRK-like family, member 3	SLITRK3	Enriched
4	mab-21-like 2 (C. elegans)	MAB21L2	Enriched
14	prostaglandin D2 receptor (DP)	PTGDR	Enriched
8	exostoses (multiple) 1	EXT1	Depleted
10	serine/threonine kinase 32C	STK32C	Depleted
12	adhesion molecule with Ig-like domain 2	AMIGO2	Depleted
*MG63*			
1	LIM homeobox 9	LHX9	Enriched
5	protocadherin beta 8	PCDHB8	Enriched
11	Wilms tumor 1	WT1	Enriched
1	potassium voltage-gated channel, member 3	KCND3	Enriched
10	serine/threonine kinase 32C	STK32C	Depleted

The chromosomal location, gene name and symbol, and Me-DIP-chip enrichment status relative to osteoblasts for U2OS and MG63 cells are indicated.

**Figure 4:**
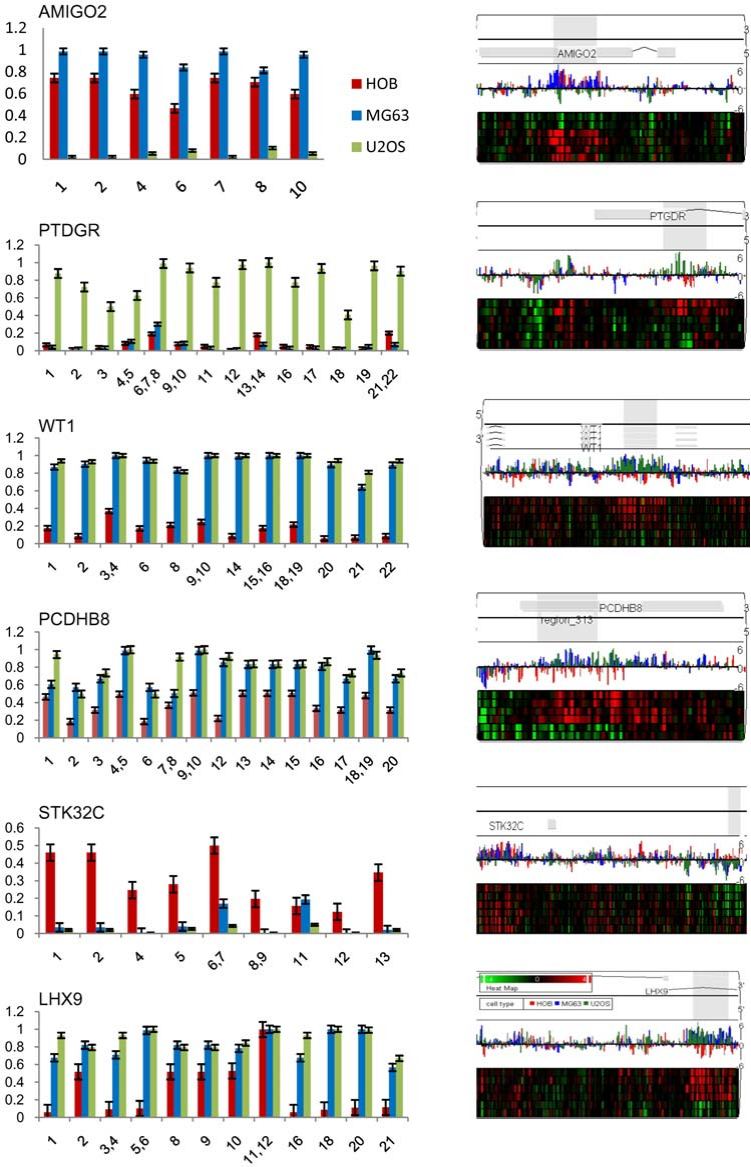
Validation of Me-DIP-chip data using quantitative DNA methylation analysis. Six genes from Table 1 were subject to EpiTYPER quantitative analysis of DNA methylation in CpG dinucleotides across 400 nucleotide regions detected as significantly enriched/depleted in Me-DIP-chip experiment. On left, the bar charts show levels of methylation (0–1–0–100%), on y-axis and individual CpG dinucleotides on the x-axis, and the corresponding error bars based on triplicate experiment. On right, the PGS-generated region views of the corresponding significantly enriched/depleted genes are labelled as in Figure 3.

In order to validate the methyl-C IP reaction, and show that Me-DIP-chip-detected enriched/depleted regions are indeed hyper-/hypomethylated at their corresponding CpGs, detailed quantitation of CpG methylation using the EpiTYPER (Sequenom) mass-spec analysis of bisulfite converted DNA was performed. The six regions analysed belonged to genes that were detected as differentially enriched by both Me-DIP-chip (Table S3, and S4) and gene-specific real-time PCR (Table 1). *AMIGO2* was significantly depleted and *PTDGR* significantly enriched in U2OS cells only. *WT1*, *PCDHB8*, and *LHX9* were significantly enriched and *STK32C* was significantly depleted in both U2OS and MG63. Quantitative analysis of DNA methylation across all these genes confirmed both Me-DIP-chip and real-time data (Figure 4). Detailed analysis of these data shows that our Me-DIP-chip approach is capable of detecting both robust and more subtle hypomethylation and hypermethylation events (compare *AMIGO2* with *STK32C*, and *WT1* with *PCDHB8*). Furthermore, the primers for *LHX9* were designed to overlap the 5′end of the s-HMM region (CpGs 16–21) and extend into l-HMM region (CpGs 1–15) (Figure S2). Although majority of the CpGs are relatively hypermethylated across the regions, the relative levels of CpG 16 through 21 hypermethylation were clearly more exaggerated.

### Integration of genomic DNA methylation profiles with genome-wide expression and copy number changes

The Me-DIP-chip approach resulted in identification of hundreds of hypo- and hypermethylated gene targets in both U2OS and MG63 cells in relation to normal osteoblasts. In order to further narrow down the list of functionally relevant changes in DNA methylation (i.e. ones that may play a role in cancer-specific deregulation of gene expression) we integrated Me-DIP-chip findings with the genome-wide expression profiling. Furthermore, the studies of genomic imbalance in MG63 and U2OS cell lines in our laboratory, using FISH, SKY, mBAND, and recently a-CGH have identified many specific structural and copy number changes in these cell lines [20]–[22], [24], [25]. We analyzed the significant changes in gene copy number detected by a-CGH profiling to evaluate the relative contributions of genomic and epigenomic imbalance to gene expression changes. The integration of epigenomic, genomic imbalance and gene expression profiles was performed using PGS software.

Microarray analysis of gene expression (p<0.01, +2/−2 fold change, 2 arrays/sample) revealed significant changes in gene expression in U2OS and MG63 cells relative to normal osteoblast levels. U2OS had 1881 overexpressed, and 1400 uderexpressed genes (Table S5), while MG63 cells exhibited overexpression in 1060, and underexpression in 1072 genes (Table S6). VENN analysis of Me-DIP-chip results and expression array data revealed 106 genes that were hypermethylated and underexpressed in U2OS (Table S7), and 34 in MG63 (Table S8). Eight of the hypermethylated and underexpressed genes were common to both cell lines (Table S9). The methylation and enrichment status of one of the common genes, *LHX9*, has been interrogated and confirmed in detail (Figure 3, and 4). Alternatively, significant hypomethylation and overexpression was evident in 76 genes in U2OS (Table S10), and 92 genes in MG63 cells (Table S11).

In order to identify genes belonging to regions of significant copy number change, the results of the segmentation algorithm of a-CGH profiles were annotated with the Affymetrix HuGene-1_0-st-v1.na24.hg18.transcript.csv library. In U2OS, 371 genes were detected in significant regions of gain detected on chromosomes 7, 8, 14, 17, and 22, while 178 genes in regions of significant genomic loss on chromosomes 1, 2, 7, 8, 10, 12, 13, 19, and 21 (Table S12). There were 433 genes in regions of genomic gain in MG63 cells overlapping portions of chromosomes 1, 2, 4, 5, 6, 8, 9, 10, 16, and 19, and 171 genes in regions of significant genomic loss on chromosomes 3, 4, 6, 7, 9, 11, 13, and 14 (Table S13).

**Figure 5:**
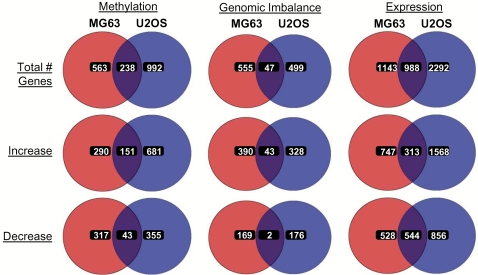
VENN analysis of gene-specific epigenetic, genetic, and gene expression changes between U2OS and MG63 cells. The lists of PGS generated genes with significant changes in DNA methylation, genomic imbalance, and gene expression, in relation to normal osteoblasts are compared using VENN analysis between the U2OS and MG63 cells.

We next analyzed the correlation in gene specific changes from the global epigenomic, genomic, and gene expression profiles between U2OS and MG63 cell lines (Figure 5). VENN analysis of genomic expression profiles showed the most consistent overlap, where nearly half of the aberrantly-expressed genes from MG63 also exhibited significant changes in gene expression in U2OS cells. Importantly, majority of these genes (857 out of 988) showed same type of change (i.e. increase or decrease) in both cell lines. In contrast, genes with genomic imbalance showed very little overlap between the two cell lines. Significantly, overlap was frequently seen in regions of genomic gain, with 43 genes belonging to the commonly gained region of chromosome 8q in both U2OS and MG63 cells. Conversely, epigenomic profiling revealed that a large proportion of genes with significant changes in DNA methylation were common to both U2OS and MG63. Out of 238 commonly affected genes, 151 genes were hypermethylated, and 43 genes were hypomethylated in both cell lines.

**Figure 6:**
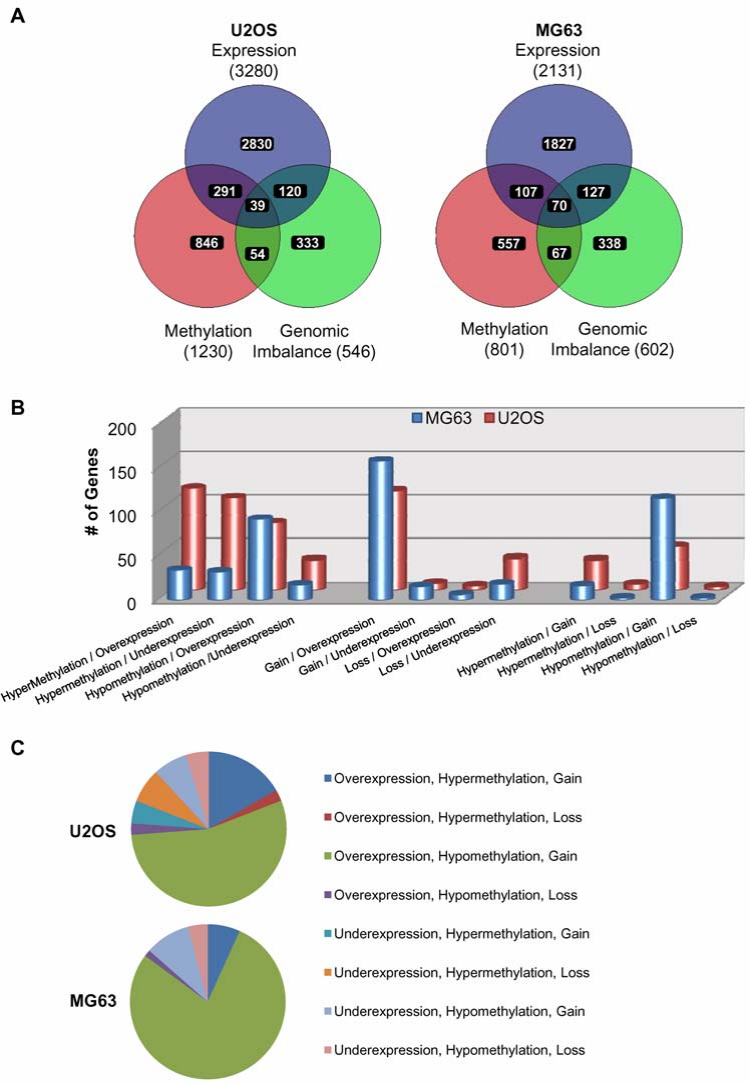
VENN analysis of gene-specific epigenetic, genetic, and gene expression changes in U2OS and MG63. (A) The lists of PGS generated genes with significant changes in DNA methylation, genomic imbalance, and gene expression, in relation to normal osteoblasts are compared using VENN analysis in U2OS and MG63 cells. (B) Analysis of the two-way intersects for the gain/loss form Figure 6A between DNA methylation and gene expression (left), genomic imbalance and gene expression (middle), and DNA methylation and genomic imbalance (right). y-axis represents the number of genes. (C) Pie chart of the 3-way intersect for the gain/loss changes from Figure 6A for DNA methylation, genomic imbalance, and gene expression.

In order to examine the global gene-specific changes in relation to epigenetic, genetic and gene expression changes within each cell line we performed 3-way VENN analysis of the significantly affected genes (Figure 6A). This analysis revealed a considerable overlap between genes affected by changes in the 2-way cross sections of either epigenetic, genetic or expression profiles. The intersects between DNA methylation and gene expression showed 330 and 177 genes; genomic imbalance and gene expression included 159 and 197; and DNA methylation and genomic imbalance 93 and 137 genes in U2OS and MG63 cells respectively. To further interrogate the status of these genes in relation to either gain or loss of DNA methylation, genomic content, or gene expression, more detailed comparisons were performed (Figure 6B). This analysis revealed that majority of genes with genomic imbalance and gene expression alterations, display genomic gain and over-expression in U2OS and MG63 cells. Furthermore genomic imbalance most strongly correlated to DNA methylation disruptions in the form of genomic gain and hypomethylation in both cell lines. The correlation between DNA methylation and gene expression showed distinct profiles in the two cell lines. In MG63 cells, large proportion of the genes with aberrant epigenetic and in gene expression profiles were hypomethylated and overexpressed, while in the U2OS cells in addition to hypomethylation and overexpression, large number of genes were affected by hypermethylation and underexpression, and hypermethylation and overexpression. Examination of the genes belonging to the 3-way intersect in Figure 6A revealed that majority of the genes affected by epigenetic, genetic, and gene expression changes were hypomethylated, gained and overexpressed in both U2OS and MG63 cells (Figure 6C). Tables with the lists of these genes, their relative methylation levels, expression values, and genomic copy number status for U2OS (Table S14) and MG63 (Table S15) include genes that we have previously identified in the common regions of gain in these cell lines including regions in chromosome 8q [20]–[22], [24], [25]. One of such genes in MG63 cells was the *c-MYC* oncogene.

### Gene network analysis

**Figure 7:**
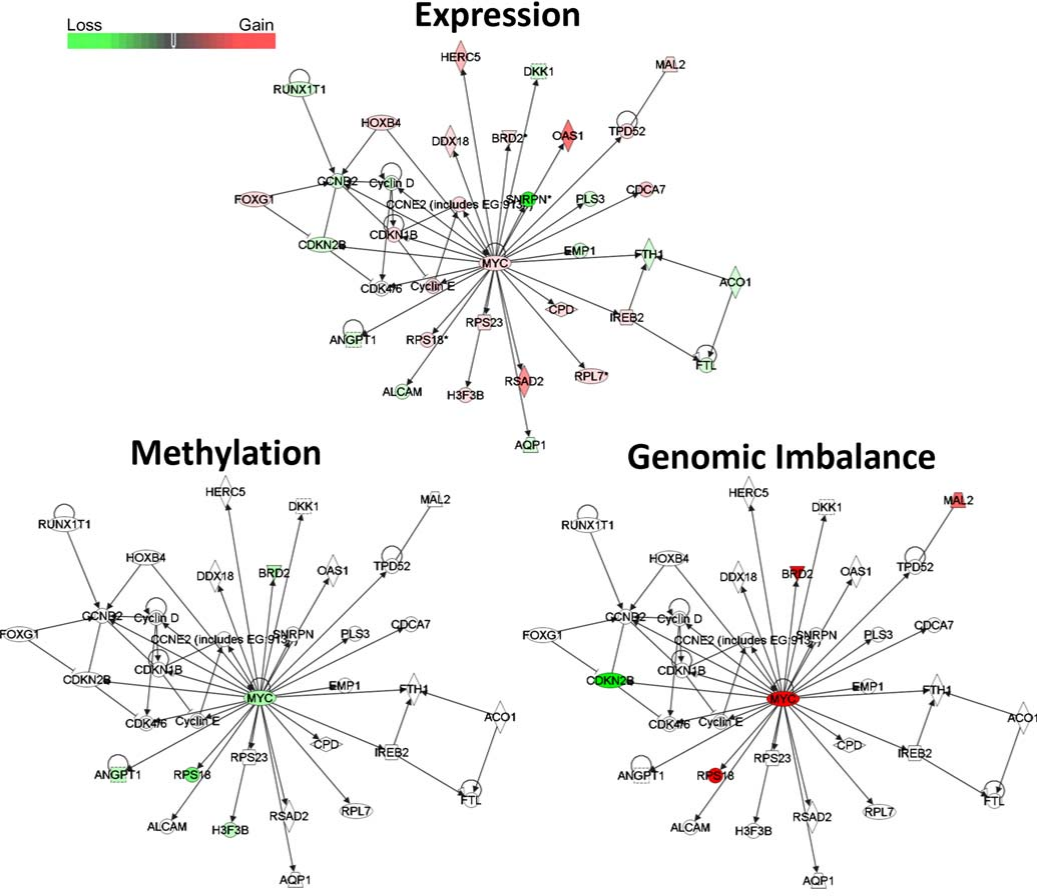
*MYC* network-related changes in gene expression, DNA methylation, and genomic imbalance in MG63 cells. IPA analysis of gene expression, DNA methylation , and genomic imbalance changes in *MYC* oncogene related pathways. Red denotes gain, and green loss of the corresponding variable.

In order to identify gene networks with the disrupted expression profiles in U2OS and MG63 cells, relative to normal osteoblasts, and the possible epigenetic and genetic contributions to these networks, we performed Ingenuity Pathway Analysis (IPA) of the genes with aberrant expression profiles. This analysis revealed that four out of five most significantly affected biological functions were common to U2OS and MG63, and included cellular movement, cellular growth and proliferation, cell to cell signalling and interaction, and cell death (Figure S3). Furthermore, comparative analysis of this dataset revealed a significant contribution of genes with significant changes in DNA methylation and genomic imbalance to all of these biological functions (Figure S3). The top three networks with most significant changes in gene expression in U2OS and MG63 cells were associated with cancer-related disruptions in cell cycle, proliferation, gene expression, and cell death (Table S16). One of these networks that was identified in MG63 cells centers around the *c-MYC* oncogene and includes genes involved in cellular function and maintenance, small molecule biochemistry, and cancer (Figure 7). Four out of five hypomethylated genes, including *c-MYC*, were also overexpressed in this pathway. Furthermore, four genes showing genomic gain, including *c-MYC*, were also overexpressed, while the only gene showing significant loss, the tumour suppressor *CDKN2B*, was also underexpressed.

**Figure 8:**
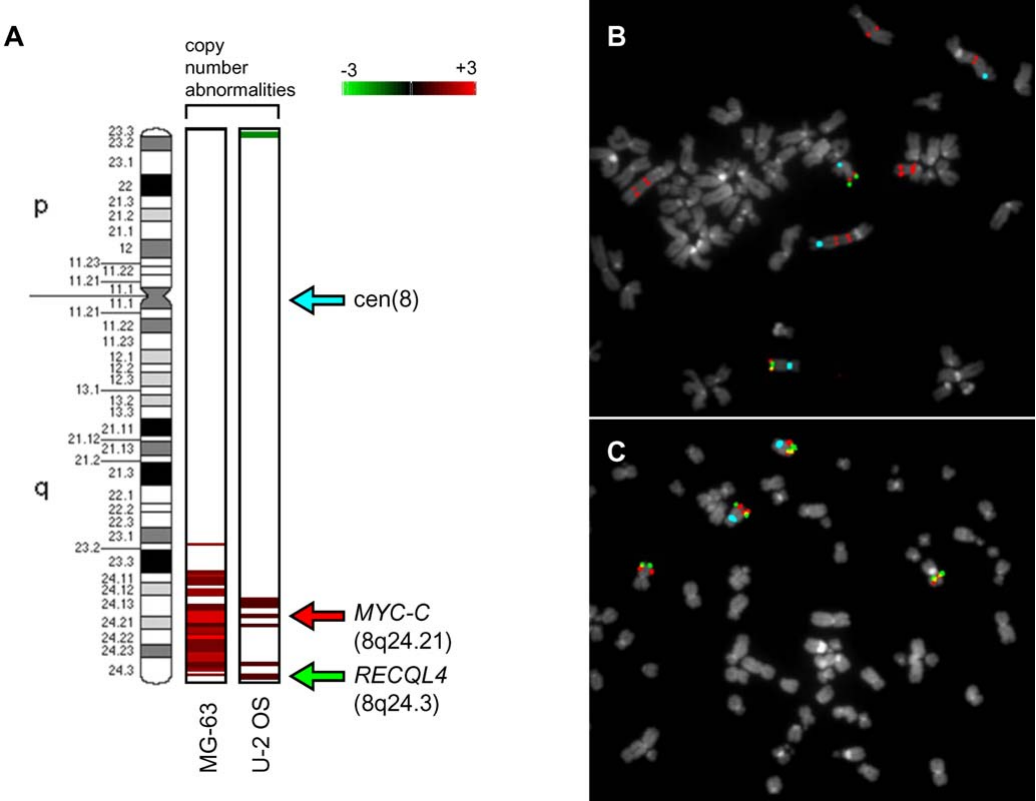
Validation of array-CGH abnormality calls by metaphase FISH. A: the copy number abnormality calls identified by array-CGH analysis are shown on the left side of the chromosome 8 ideogram (850-band resolution) for each cell lines. The log_2_ ratios of a-CGH enrichment detected by genomic segmentation algorithm are represented by a spectrum from green (−3) to red (+3). Arrows indicate the chromosomal localization of the FISH probes. Metaphases from MG-63 (B), and U-2 OS (C), were co-hybridized with the following probes: chromosome 8 centromere (pale blue), RP11-440N18 (8q24.21) (red) and RP11-349C2 (8q24.3) (green).

To further analyse the copy number status of the *c-MYC* oncogene FISH was performed. In order to provide additional validation our a-CGH data we analysed another gene located near the telomere of 8q, the damage repair-associated *RECQL4*, which was detected as significantly enriched in U2OS, but not MG63 cells. Figure 8A shows the results of PGS genomic segmentation analysis in U2OS and MG63 cell lines, revealing a high-level gain of *c-MYC* in MG63 and no significant change in *RECQL4*, and low-level gains in *c-MYC* and *RECQL4* in U2OS cells. FISH analysis showed on average ten copies of *c-MYC* and only two copies of *RECQL4* in MG63 (Figure 8B), with average four copies of both *c-MYC* and *RECQL4* in U2OS cells (Figure 8C), thereby confirming our a-CGH data for this region.

## Discussion

Both epigenetic and genetic changes contribute to development of human cancer. In this paper we describe an integrative approach for analysis of the cumulative effects of genome-wide changes in DNA methylation, genomic imbalance, and gene expression in OS cell lines relative to the normal human osteoblasts. The identification of differential methylation in the originally described Me-DIP-chip protocol involves detection of enriched regions in the IP fraction relative to the IN fraction within a particular sample, and subsequent comparison between the samples [37], [38]. Although this approach resulted in successful mapping of genome-wide DNA methylation profiles it suffered from some limitations, including low resolution arrays, as well as an indirect approach for detection of differentially methylated genes between cell lines.

We utilized Affymetrix Promoter Tilling Arrays that were previously used for high-resolution mapping of epi-toxicogenomic profiles of global histone acetylation patterns in a breast cancer model of environmental exposures to a carcinogen benzopyrene [39]. By applying this Me-DIP-chip protocol, including specific HMM-based detection algorithms on arrays, significant changes in DNA methylation in regions as short as 300 nucleotides were detected (Table S1 and S2) (Figure 4). This is a particularly important finding, given the evidence that changes in DNA methylation profiles of very short spans of DNA in gene promoters can significantly affect gene expression [40]–[42]. Such high resolution analysis also allowed for a precise detection of differentially methylated regions within gene promoters (both hypo- and hypermethylation), as well as highly efficient design of gene-specific validation experiments (Figure 2) including gene-specific real-time PCR for Me-DIP enrichment (Figure 3), and EpiTYPER quantitative methylation analysis (Figure 4) across many gene promoters.

Region detection in Me-DIP-chip or ChIP-chip studies are commonly performed by detecting enriched regions by applying a chosen “cut off” for the IP/IN signal and subsequent comparison of the enriched regions between cell lines [37]–[39], [43]. This indirect approach is limited to detection only enriched regions (i.e. hypermethylated), and may be biased, based on the CpG density for specific regions [38]. To address these limitations, the detection of significant differentially enriched regions was based on the application of the HMM algorithm on the fold differences between IP/IN-normalized signal of the cancer (U2OS, MG63) versus normal (human osteoblast) cells across each of the 4.2 milion probes individually. Therefore, by making detection of significance relative to the normal background, our approach was designed to detect cancer-specific hypo- or hypermethylation in OS cells.

Cancer-specific changes in DNA methylation may play a role in many cellular functions including destabilization of chromatin, promotion of genomic instability, and hypomethylation of repetitive elements, while some changes may play a functional role in deregulation of gene expression [3], [6], [44]. Affymetrix Promoter Tilling arrays are annotated to 25,500 human genes, allowing integration of OS gene-specific methylation profiling with the expression microarray data. VENN analysis of the lists of genes with cancer-specific DNA methylation and gene expression signatures narrowed down the list of genes of interest, to 106 genes that were hypermethylated and underexpressed in U2OS (Table S7), and 34 in MG63 (Table S8). Genes common to both cell lines (Table S9), included *LHX9* that was interrogated and confirmed in detail (Figure 3, and 4). Significant hypomethylation and overexpression was evident in 76 genes in U2OS (Table S10), and 92 genes in MG63 cells (Table S11). These data suggest that, in addition to the hypermethylation and possible loss of gene expression, increases in gene expression driven by hypomethylation may play a role in OS.

*c-MYC* was shown to be hypomethylated in acute leukemia derived from myelodysplastic syndromes [45]. In a recent study it was shown that hypomethylation of the LINE 1 retrotransposon, as well as amplification of *c-MYC* can be used to predict tumour stage in prostate cancer [46]. Our data show hypomethylation, genomic gain, and overexpression of *c-MYC*-oncogene that is particularly evident in MG63 cell line (Table S15) (Figure 8). Furthermore, the IPA analysis of cancer-specific changes in gene expression networks of MG63 cells revealed significant disruption in the *MYC*-centered gene expression network, and revealed both genetic and epigenetic abnormalities in a number of genes in this pathway (Figure 7).

Majority of the genes that showed significant changes in DNA methylation, genomic imbalance, and gene expression exhibited DNA hypomethylation, genomic gain and overexpression in both U2OS and MG63 cell lines (Figure 5C). Furthermore, majority of these genes (Table S14 and S15) are located in the regions we and others have previously shown to be amplified in OS, including chromosomes 6p, 8q, 9p, and17p in OS tumours and cell lines [18]–[25]. These data are consistent with the emerging evidence in other cancers that links DNA hypomethylation and regions of genomic imbalance and genomic instability [47]–[50].

Regions of hypo- and hypermehylation in U2OS and MG63 cells mapping close to larger repeat elements including segmental duplications (>1000 nucleotide) were observed. For example we identified a large region located at chromosome 8q21.2 spanning genes *REXO1L1* and *REXO1L2P* that exhibited DNA hypomethylation in U2OS and not MG63 or osteoblast cells (Figure S4). Interestingly, this hypomethylation spanned 8 CpG islands that overlapped regions of segmental duplications, while outside CpG islands were not affected. Alternatively, we also observed large regions of hypermethylation including one at chromosome 5q31spanning nearly 2 Mb and including three Protocadherin gene families, in both U2OS and MG63 cells (Figure S5). Similarly, this hypermethylation was evident in the high-density CpG island cluster that mapped at and around segmental duplication hotspot. We validated one of these genes, *PCDHB8* using real-time PCR (Figure 3) and quantitative DNA methylation analysis (Figure 4). These observations indicate that in addition to gene specific hypo- and hypermethylation changes, disruptions of DNA methylation profiles may affect large regions of DNA which in some cases may be associated with genomic repeats.

Genomic hypermethylation, especially in the context of tumour suppressor inactivation may present an attractive target for the chemotherapeutic intervention. In our previous study we have performed microarray analysis of gene expression in U2OS cells treated by the demethylating agent Decitabine and identified genes with significant Decitabine-dependent overexpression [35]. Comparison of that dataset with the genes that were differentially methylated in our Me-DIP-chip analysis of U2OS cells revealed 43 genes that were in common, majority of which (32) were hypermethylated in U2OS relative to normal osteoblasts (Table S17). One of those genes, *PTGDR*, exhibited 14-fold hypermethylation in U2OS, and 6.5-fold overexpression after Decitabine treatment of U2OS cells. Our validation of the enrichment (Figure 3), and CpG methylation (Figure 4) have confirmed extensive hypermethylation of *PTGDR* in U2OS cells specifically. Interestingly, a recent paper by Sugino and coworkers showed hypermethylation and Decitabine-induced overexpression of the *PTDGR* and its' alternative transcript *PTDGR2* in neuroblastoma, which is another common paediatric solid tumour [51].

In addition to gene hypermethylation, we observed promoter related hypomethylation in 397 and 359 genes in MG63 and U2OS cells respectively (Table S3, and S4), 19 and 25 percent of which showed overexpression profiles relative to normal osteoblasts respectively (Table S7 and S8). Such correlation between hypomethylation and overexpression is particularly evident in the MG63 cell line (Figure 6B). Good agreement between hypomethylation and overexpression was also evident in U2OS cells, as was hypermethylation with underexpression. A subset of genes also exhibited hypomethylation with underexpression (Figure 6B). The evidence of genes with concurrent hypomethylation and underexpression in U2OS may be related to additional factors such as presence/absence of specific transcription factors, epigenetic histone modification, etc. In both U2OS and MG63 cells hypomethylation seems to also strongly correlate with genomic gain, while genomic gain also correlates with gene overexpression (Figure 6B). Another important observation is that in U2OS cells 7.5% (93/1230) differentially methylated genes were in the regions of copy number change, while in MG63 cells these genes comprise 17.1% (137/801) (Figure 6A). This marked difference between the two cell lines is further evident in the analysis of the types of methylation changes in the regions of genomic imbalance. While majority of such genes in MG63 cells are hypomethylation events in the regions of genomic gain, in U2OS cells both gene hypomethylation and hypermethylation are evident in the regions of genomic gain (Figure 6B). In both cell lines majority of epigenetic changes in regions of genomic imbalance is regions of genomic gain. One possible explanation for this observation is that unlike in regions of genomic loss where gene expression or dosage will be directly affected by the loss of genomic content, in the regions of genomic gain DNA methylation may provide an additional “layer” of control of the gene expression. As such epigenetic changes in the regions of genomic gain may have an additional selective advantage during tumour evolution. Furthermore, 3-way VENN analysis also shows that a subset of genes with epigenetic, genetic and gene expression changes predominantly display hypomethylation, genomic gain, and overexpression, in both U2OS and MG63 cells (Figure 6C). This observation suggests the possibility that during tumour evolution, hypomethylation and gain of certain genomic regions may act cooperatively to increase the gene expression/gene dosage levels. It is important to note that although Affymetrix Promoter Arrays that were used in this study provide detailed information of extensive promoter regions of more than 25,500 genes, additional epigenetic changes in intragenic regions and genomic repeats may account for additional epigenetic influences in these cell lines.

To further assess the contribution of epigenetic and genetic changes to the gene expression profiles in U2OS and MG63 cells we performed Ingenuity Pathway Analysis of gene networks and cellular functions with most significant changes in gene expression, and compared it to the analyses of the DNA methylation and genome imbalance in these cells. Although U2OS and MG63 cells bear significant morphological and genetic differences (for example U2OS is P53-positive, MG63 is P53-negative cell line), top four out of five most significantly-affected cellular functions are identical between the two cell lines (Figure S3). There is also a significant epigenetic and genetic contribution to these cellular functions. Such correlation is also seen in a 2-way VENN comparison of epigenetic, genetic, and gene expression changes in these two cell lines (Figure 5). Interestingly, in U2OS there was a strong epigenetic contribution to disruption of cellular growth and proliferation, while in MG63 cells there is a robust genetic contribution to deregulation of these cellular functions, as well as cellular development (Figure S3). An example of a gene network with evidence of potential genetic and epigenetic contribution to the cancer-specific gene expression profile is the *c-MYC*-related network in MG63 cells (Figure 7 and 8). Collectively, these data suggest that both epigenetic and copy number disruptions play a cumulative role in deregulation of gene networks in OS cell lines.

Integration of genomic and epigenomic studies promises to provide us with new insights in cancer aetiology. Recently, genome-wide integrative studies of epigenetic and gene expression profiles in Arabidopsis [52] and human leukemia [53] described correlation between epigenomic alterations and gene expression profiles. In this paper, we have used an integrative epigenetic, genome imbalance, and gene expression analysis to provide first genomic DNA methylation maps, and identified and validated genes with aberrant DNA methylation in two osteosarcoma cell lines. In conclusion, these data provide evidence of the cumulative roles of epigenetic and genetic mechanisms in cancer-related deregulation of gene expression networks.

## Materials and Methods

### Cell culture and DNA & RNA extraction

The human OS cell lines U2OS (ATCC # HTB-96) and MG63 (ATCC # CRL-1427 ) were purchased from American Type Culture Collection ATCC (Rockville, MD) and maintained in alpha-Minimum Essential Medium (alpha-MEM) supplemented with 10% heat inactivated Fetal Bovine Serum and 2 mM L-Glutamine. Normal human osteoblasts are primary osteoblasts from the hip bone of a normal male donor that were purchased from PromoCell (Heidelberg, Germany, Catalogue # C-12760) and maintained in medium provided by the manufacturer and used at culture passage 3. Three days after plating (∼80% confluent), cells where harvested for DNA or RNA extractions.

DNA was extracted after harvesting the cells by trypsinization followed by phenol-chloroform extraction and subsequent precipitation in 100% ethanol. DNA precipitate was washed with 70% ethanol then eluted in DNAse free water. Total RNA from U2OS, MG63 cells and osteoblasts was extracted using the TRIzol reagent according to the manufacturer's instructions (Invitrogen), and analyzed for quantity and quality using bio-analyzer (Agilent Technologies, USA).

### Me-DIP-chip

Analysis of genomic methylation profiles across 25,500 gene promoters (2.5 Kb 3′, and 7.5–10 Kb 5′ from the transcriptional start site) at 35 nucleotide resolution was performed by methylated DNA immunoprecipitation followed by the microarray hybridization (Me-DIP-chip) using the Affymetrix Human Promoter 1.0R Tilling Arrays using a modification of the Affymetrix chromatin immunoprecipitation assay protocol. Genomic DNA from normal human osteoblasts, U2OS and MG63 cells was sonicated (Sonic Dismembrator Model 100, Fisher Scientific) to reduce the size of DNA fragments to 200–1000 nucleotides and used as input (IN). DNA (4 µg) was immunoprecipitated (IP) with 10 µl of 5 mC Antibody (Eurogentec, BI-MECI-0500) using the Me-DIP protocol [37], with following modifications: The antibody-DNA complexes were immunoprecipitated using Protein-A Agarose Beads (Upstate, Massachusetts, USA , Catalogue # 16-125), and the recovered DNA was purified using the Qiaquick PCR purification kit (Qiagen, #28106, Maryland USA).

Random priming reactions of total 50 ng of IP and IN DNA, followed by the genomic PCR, were performed using a modification of the Affymetrix chromatin immunoprecipitation assay protocol as previously described [39]. The uracil glycosilase treatment, streptavidin/phycoerythrin labeling, hybridization and microarray scanning were performed as per Affymetrix chromatin immunoprecipitation assay protocol at The Centre for Applied Genomics (The Hospital for Sick Children, Toronto, ON, Canada). All microarray experiments were performed in duplicate for both IP and IN fractions of each DNA sample starting with initial sonication step; totalling 4 arrays per DNA sample (2 IP and 2 IN).

### Array-CGH (a-CgH)

The Agilent Human Genome CGH microarray 44k and 244A (Agilent Technologies, Inc., Palo Alto, USA) were used for the MG63 and U2OS array-CGH experiments, respectively. Three µg of Human Genomic DNA from multiple anonymous male donors (Promega Corporation, Madison, USA) and 3 µg of test genomic DNA sample were subject array-CGH as previously described [29]. Arrays were washed according to the manufacturer's recommendations; air dried, and scanned using an Agilent 2565AA DNA microarray scanner (Agilent Technologies, USA), and processed using Agilent Feature Extraction software. Dye-swapped duplicate experiments were carried out for both MG63 and U2OS cell lines.

### Expression Arrays

Genomic RNA expression analysis was performed using the Affymetrix Gene 1.0 ST arrays, where each of the 28,869 genes is represented on the array by approximately 26 probes spread across the full length of the gene, providing a more complete and more accurate picture of gene expression than 3′ based expression array designs. RNA (200 ng) from normal human osteoblasts, U2OS and MG63 cells was analyzed as per manufacturer's instructions at The Centre for Applied Genomics (The Hospital for Sick Children, Toronto, ON, Canada). Each microarray experiment was performed in duplicate.

### Data Analysis and Integration

Data from Me-DIP-chip and RNA expression array experiments in the form of .cel files (GCOS 1.3 software), and a-CGH .txt files (Agilent Feature Extraction software) were imported into, analysed and integrated using the Partek Genomic Suite Software (PGS) (GEO accession numbers: GSE11416, GSE7077) (Figure 1).

The Me-DIP-chip .cel files for osteoblasts, U2OS and MG63 (2 IP and 2 IN) were log_2_ transformed, normalized and imported into PGS as previously described [39]. We baseline normalized the signal using the matched-pair normalization tool in PGS, by subtracting log_2_ IN signal intensity at each of 4.2 million from cell type-matched IP signals resulting in 6 corrected datasets (IP1 cor. and IP2 cor. for each cell line). In order to determine the relative enrichment of IP cor. signals in U2OS or MG63 relative to osteoblasts, we used the PGS 1-way ANOVA tool and calculated the fold change using the geometric mean (for log-transformed data). Thus generated signals represented baseline-normalized, osteoblast-relative, and cancer cell-specific enrichment levels for each of 4.2 million probes. Significant region detection (both enrichment/hypermethylation and depletion/hypomethylation) was performed using Hidden Markov Model (HMM) tool in PGS by applying it to the fold change data for both U2OS and MG63. In order to capture significant differences in enrichment in each cancer cell line vs. normal osteoblast across shorter genomic regions (min. ∼350 nucleotides) with robust enrichment differences as well as intermediate (min. ∼500 nucleotides) and large regions (min. 1400 nucleotides), three HMM algorithms were applied (s-HMM, m-HMM, and l-HMM). Following cut-offs were used: s-HMM (min. probes: 10, detection states: −5,5, ignore state: 0, max. probability: 0.99, genomic decay: 10,000, sigma: 2), m-HMM (min. probes: 15, detection states: −3,3, ignore state: 0, max. probability: 0.99, genomic decay: 10,000, sigma: 1), and l-HMM (min. probes: 40, detection states: −1.5,1.5, ignore state: 0, max. probability: 0.99, genomic decay: 10,000, sigma: 1). Significantly enriched/hypermethylated and depleted/hypomethylated HMM regions were annotated to the corresponding genes present on the Affymetrix Gene 1.0 Array using the HuGene-1_0-st-v1.na24.hg18.transcript.csv file. The visualization of data using heat maps, .wig files for UCSC Genome Browser, genome view files, dot plots, and VENN diagrams and corresponding data tables/lists was performed using PGS as previously described [39].

The expression array .cel files for osteoblasts, U2OS, and MG63 (2 arrays each) were imported using PGS Gene Expression Workflow tool (subject to RMA normalization and log_2_ transformation). The significantly over- and under-expressed genes in U2OS and in MG63 cell lines compared to normal osteoblasts were detected using 1-way ANOVA tool at p<0.01 and +/− 2-fold enrichment. Significantly over- and under-expressed regions were annotated to the corresponding genes present on the Affymetrix Gene 1.0 Array using the HuGene-1_0-st-v1.na24.hg18.transcript.csv file. Visualizations and VENN analysis were performed in PGS.

To analyze genomic imbalance in U2OS and MG63 (2 arrays each) the processed R signal and processed G signal columns from the Agilent Feature Extraction-generated a-CGH .txt files were imported into PGS. The cancer cell line-specific signal across all probes was normalized as a ratio to baseline using Normalise to Baseline Tool in PGS, where baseline data corresponded to the normal human DNA. The data was then log_2_ transformed using the PGS Normalization and Scaling Tool. In order to detect regions of genomic gain and loss we applied the Genomic Segmentation tool with segmentation parameters set at: min. probes: 10 for MG63, and 50 for U2OS (due to 5-fold higher probe density at respective arrays), p-value threshold: 0.01, and signal to noise: 0.1. Region report was set at values bellow −1/+1 (log_2_) and p-value threshold of 0.05. Regions of significant gain or loss were annotated to the corresponding genes present on the Affymetrix Gene 1.0 Array using the HuGene-1_0-st-v1.na24.hg18.transcript.csv file. Visualizations and VENN analysis were performed in PGS.

The integration of significantly hypo- and hyper-methylated, over- and under-expressed, and gained and lost gene lists was performed using the VENN tool in PGS, and visualization to the resulting tracks was performed as previously described [39].

### Network identification and canonical pathway analysis

Functional identification of gene networks was performed using Ingenuity Pathway Analysis program as previously described [39].The tables representing the differentially expressed, methylated and genomicaly imbalanced genes from U2OS and MG63 cells as well as the corresponding expression (relative to osteoblasts), methylation enrichment (mean of regions associated with individual gene, relative to osteoblasts ), and gain (+1) and loss (−1) values were imported as individual experiments using the Core Analysis tool. The analysis was performed using Ingenuity Knowledge Database and was limited to direct interactions only.

### Gene-specific real-time PCR validation of Me-DIP DNA enrichment

In order to validate the enrichment status of genes detected by Me-DIP-chip array we performed a gene specific real-time expression analysis in a set of both common and cell-line specific significantly enriched and depleted genes in MG63 and U2OS cells. Real-time PCRs were performed in 1X SYBR Green PCR mixture (Bio-Rad, USA), 10 ng of the original IP or IN DNA, and 1 µM gene-specific primers (Table S18) that were designed using Primer 3 (http://frodo.wi.mit.edu/) software. The reactions were performed in triplicate and relative enrichment determined as a ratio of U2OS or Mg63 IP(Ct)/IN(Ct) over osteoblast IP(Ct)/IN(Ct).

### EpiTYPER quantitation of CpG Methylation

In order to validate the methylation status in a set of a genes detected as enriched or depleted in the Me-DIP-chip experiment we performed the quantitative analysis of CpG using the EpiTYPER System for quantitative DNA methylation analysis using the MassARRAY system (Sequenom, USA) at the Analytical Genetics Technology Centre (Princess Margaret Hospital, Toronto, ON, Canada), as per manufacturer's instructions (http://www.analyticalgenetics.ca/Function/Business/Service/Methylation.aspx), using the original DNA samples for U2OS, MG63 and human osteoblasts that were used in the Me-DIP-chip experiment. The gene-specific bisulfite primers (Table S18) were designed to overlap HMM-detected regions of differential enrichment using the MethPrimer Software (http://www.urogene.org/methprimer/index1.html). Each analysis was performed in triplicate and all resolvable CpG signals were mapped and standard error bars are displayed.

### Fluorescence In-Situ Hybridization (FISH)

Metaphase spreads for cytogenetics analysis were prepared from the MG-63 and U-2 OS cultures using the conventional methods [54]. A commercial probe for centromere 8 was used according to the manufacturer's instructions (CEP 8 SpectrumAqua Probe, Abbott Molecular, Des Plaines, IL). BAC probe located within the 8q24.21 and 8q24.3 regions, were identified using the Resources for Molecular Cytogenetics website (www.biologia.uniba.it/rmc/). The BAC clones RP11-440N18 and RP11-349C2 were obtained from the Centre for Applied Genomics (Toronto, ON, Canada) and labeled by nick-translation (Nick-Translation Kit, Abbott Molecular) using SpectrumOrange, or SpectrumGreen (Abbott Molecular). Standard FISH procedures were followed [55]. Sides were observed using an epifluorescence Zeiss Imager Z1 microscope equipped with a digital camera Axio Cam MRm and AxioVision 4.3 capturing software (Zeiss, Toronto, ON, Canada).

## Supporting Information

Figure S1 Clustering analysis of the Me-DIP-chip array data. (A) Raw, imported .cel file data of 4.2 million probes was subject to Euclidean hierarchical clustering using PGS before (left panel) and after background normalization. The replicate experiments are numbered. Note the reproducibility of the signal in replicate experiments, and separate clustering between IP and IN arrays, as well as the separate clustering between cancer (U2OS, MG63) and normal (osteoblast) cells. (B) Principal Component Analysis (PCA) plot generated in PGS of the .cel file data revealing separate clustering similar to hierarchical clustering, totalling 28% variability across data
(7.83 MB TIF)

Figure S2 Hidden Markov Model detection of significantly enriched/depleted regions in Me-DIP-chip data. The heat-map and profile images of the LHX9 gene promoter are as described in Figure 2. LHX9 promoter in U2OS (A) and MG63 (B) exhibiting significantly enriched regions (shaded boxes) detected by both l-HMM algorithm (left), and s-HMM (algorithm). Note that l-HMM detects longer regions with overall less robust enrichment, that may include shorter regions with more robust enrichment detected by s-HMM.
(4.73 MB TIF)

Figure S3 Gene expression, epigenetic, and genetic contribution to cellular function disruption in OS. Top 5 most significantly affected biological functions in relation to the deregulation of gene expression in U2OS and MG63 versus normal osteoblasts were detected using the Ingenuity Pathway Analysis (dark blue bars), and compared to IPA analysis of DNA methylation (light blue bars) and genomic imbalance (turquoise bars) in these cells. The p-value threshold is set at 0.05.
(7.98 MB TIF)

Figure S4 Regional hypomethylation in U2OS cells. Top panel is the PGS-generated region view of the hypomethylated genomic region in U2OS cells located at 8q21.2, featuring the colour-coded profile of the signal from each cell type, and the corresponding heat-map of the replicate array experiments bellow (in log2). Middle panel shows the PGS-generated .wig file of this region imported into UCSC Genome Browser, displaying the corresponding gene, CpG island, and segmental duplication tracks.
(10.31 MB TIF)

Figure S5 Hypermethylation of Protocadherin gene family in U2OS and MG63 cells. Top panel is the PGS-generated region view of the 2 Mb hypermethylated genomic region in U2OS and MG63 cells located at 5q31.3, featuring the colour-coded profile of the signal from each cell type, and the corresponding heat-map of the replicate array experiments bellow (in log2). Middle panel shows the PGS-generated .wig file of this region imported into UCSC Genome Browser, displaying the corresponding gene, CpG island, and segmental duplication tracks.
(15.60 MB TIF)

Table S1 Significant differentially-methylated regions in U2OS cells. Enriched and depleted regions detected by the l-HMM, m-HMM, and s-HMM algorithm in U2OS cells relative to the normal osteoblast levels are shown. Table also includes identifiers such as precise chromosome location of the region and the annotated gene, gene assignment, mean enrichment level across the region (fold enrichment), region length, PGS region ID, and HMM state (l-HMM - +/− 1.5, m-HMM +/− 3, s-HMM +/− 5).
(0.42 MB XLS)

Table S2 Significant differentially-methylated regions in MG63 cells. Same as Table S1, for MG63 cells.
(0.31 MB XLS)

Table S3 Significant differentially-methylated genes in U2OS cells. Tabulation of hyper- and hypomethylated genes generated from Table S1. In case where more than one HMM algorithm is detected in a specific gene promoter, the longest HMM region is listed.
(0.30 MB XLS)

Table S4 Significant differentially-methylated genes in MG63 cells cells. Same as Table S3, for MG63 cells.
(0.20 MB XLS)

Table S5 Differentially expressed genes in U2OS cells. Genes with significant changes in expression, both over and under, relative to normal osteoblasts, and the corresponding p-value and fold change are shown.
(0.65 MB XLS)

Table S6 Differentially expressed genes in MG63 cells. Same as Table S5, for MG63 cells.
(0.43 MB XLS)

Table S7 Hypermethylated and underexpressed genes in U2OS cells. Genes with significant hypermethylation and loss of expression, relative to normal osteoblasts, and the corresponding p-value and fold change underexpression and methylation mean of region (fold enrichment) are shown.
(0.03 MB XLS)

Table S8 Hypermethylated and underexpressed genes in MG63 cells. Same as Table S7, for MG63 cells.
(0.02 MB XLS)

Table S9 Common hypermethylated and underexpressed genes in U2OS and MG63 cells. Common genes with significant hypermethylation and loss of expression in U2OS and MG63 cells, relative to normal osteoblasts, and the corresponding p-value and fold change underexpression and methylation mean of region (fold enrichment) are shown.
(0.02 MB XLS)

Table S10 Hypomethylated and overexpressed genes in U2OS cells. Genes with significant hypomethylation and gain of expression, relative to normal osteoblasts, and the corresponding p-value and fold change underexpression and methylation mean of region (fold enrichment) are shown.
(0.03 MB XLS)

Table S11 Hypomethylated and overexpressed genes in MG63 cells. Same as table S10, for MG63 cells.
(0.03 MB XLS)

Table S12 Genes in regions of significant gain and loss in U2OS cells. Precise chromosomal location and gene assignment for genes detected in regions of significant gain or loss detected by the PGS genomic segmentation algorithm in U2OS cells.
(0.11 MB XLS)

Table S13 Genes in regions of significant gain and loss in MG63 cells. Same as Table S12, for MG63 cells.
(0.13 MB XLS)

Table S14 U2OS genes with significant disruptions of DNA methylation, gene expression, and genomic imbalance. The genes from the 3-way intersect from DNA methylation, genomic imbalance, and gene expression VENN diagram (Figure 6A), and their corresponding expression and methylation values (relative to osteoblasts), and genomic imbalance status (gain or loss) are indicated.
(0.02 MB XLS)

Table S15 MG63 genes with significant disruptions of DNA methylation, gene expression, and genomic imbalance. Same as Table S14, for MG63 cells.
(0.03 MB XLS)

Table S16 Most significantly affected gene networks in U2OS and MG63 cells. Ingenuity Pathway Analysis-generated networks with most significant changes in gene expression in U2OS and MG63 cells relative to osteoblasts.
(0.03 MB PPT)

Table S17 Comparison of decitabine-induced re-expressed and Me-DIP-chip detected genes. VENN analysis of the Me-DIP-chip detected genes with significant changes in DNA methylation and genes with decitabine-induced overexpression [35] in U2OS cells. Fold enrichment in Me-DIP-chip (relative to osteoblasts), ford overexpression (relative to osteoblasts), and expression p-value are indicated.
(0.03 MB XLS)

Table S18 Real-time PCR and EpiTYPER validation primers. F - forward primer, R - reverse primer.
(0.03 MB XLS)
