# Supplementary figures and images for: In Vitro Analysis of Integrated Global High-Resolution DNA Methylation Profiling with Genomic Imbalance and Gene Expression in Osteosarcoma
Source: PLoS One. 2008 Jul 30;3(7):e2834. doi: 10.1371/journal.pone.0002834 (PMC2515339; doi:10.1371/journal.pone.0002834)

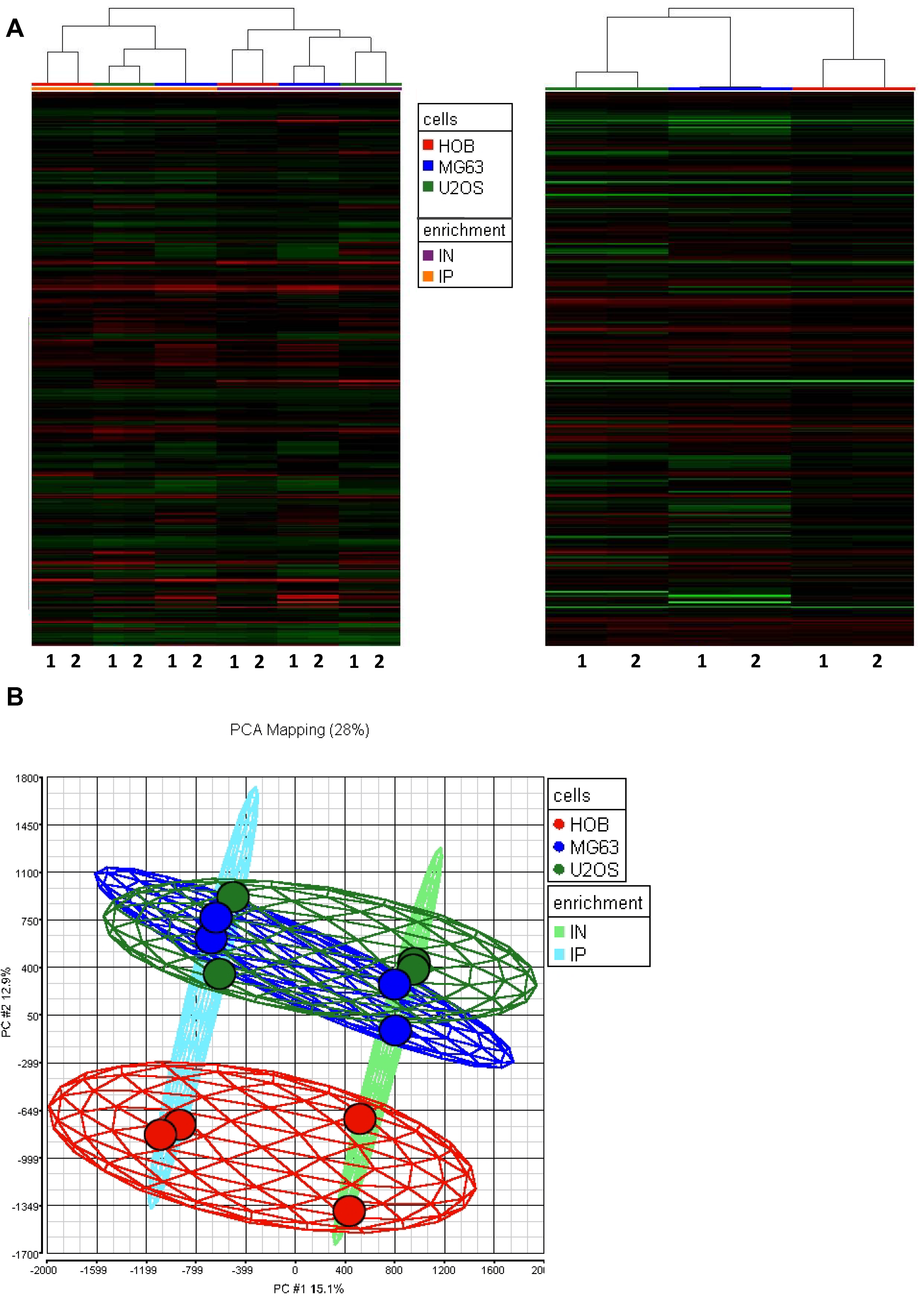

Supplement: Figure S1 — Clustering analysis of the Me-DIP-chip array data. (A) Raw, imported .cel file data of 4.2 million probes was subject to Euclidean hierarchical clustering using PGS before (left panel) and after background normalization. The replicate experiments are numbered. Note the reproducibility of the signal in replicate experiments, and separate clustering between IP and IN arrays, as well as the separate clustering between cancer (U2OS, MG63) and normal (osteoblast) cells. (B) Principal Component Analysis (PCA) plot generated in PGS of the .cel file data revealing separate clustering similar to hierarchical clustering, totalling 28% variability across data (7.83 MB TIF) [file pone.0002834.s001.png]

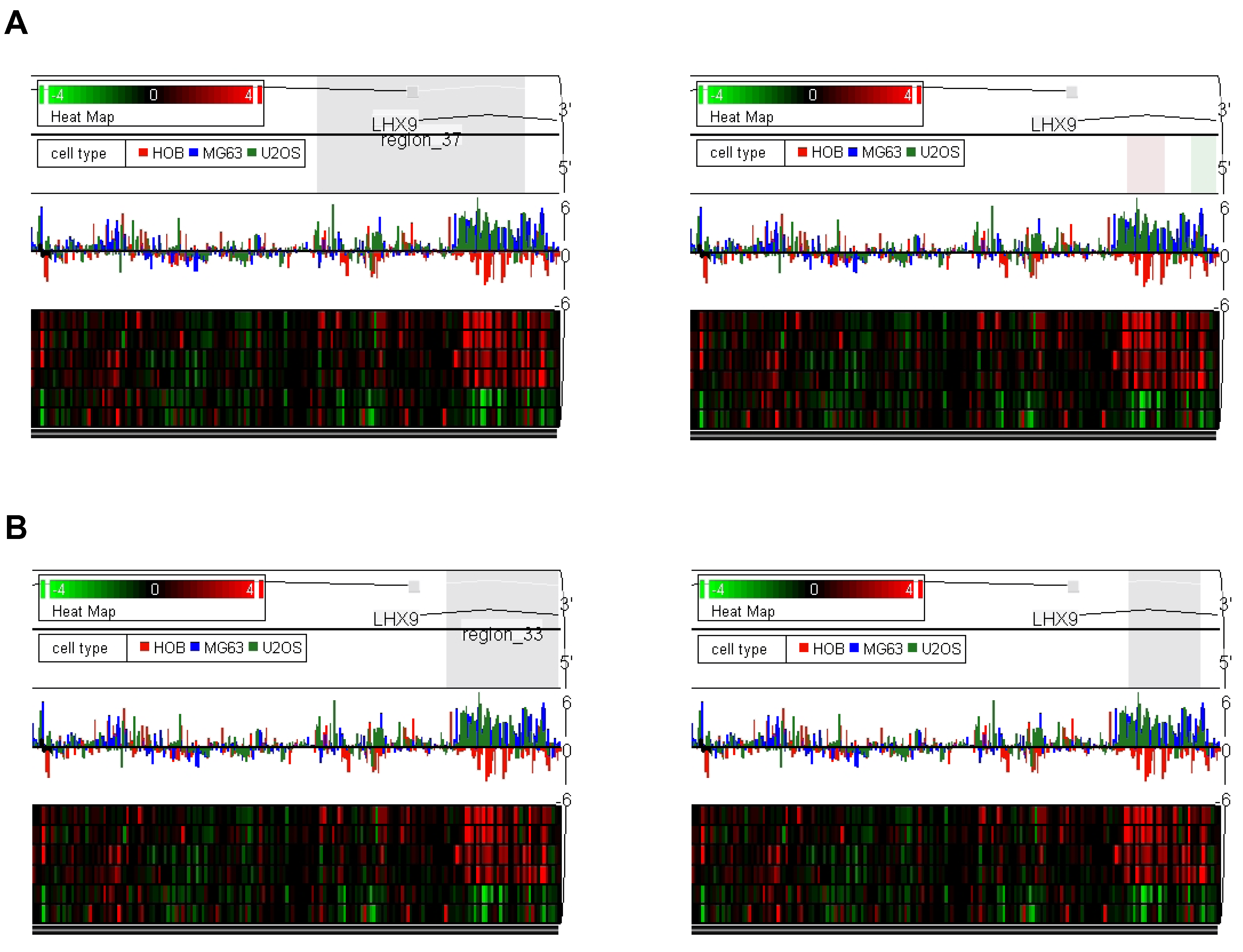

Supplement: Figure S2 — Hidden Markov Model detection of significantly enriched/depleted regions in Me-DIP-chip data. The heat-map and profile images of the LHX9 gene promoter are as described in Figure 2. LHX9 promoter in U2OS (A) and MG63 (B) exhibiting significantly enriched regions (shaded boxes) detected by both l-HMM algorithm (left), and s-HMM (algorithm). Note that l-HMM detects longer regions with overall less robust enrichment, that may include shorter regions with more robust enrichment detected by s-HMM. (4.73 MB TIF) [file pone.0002834.s002.tif]

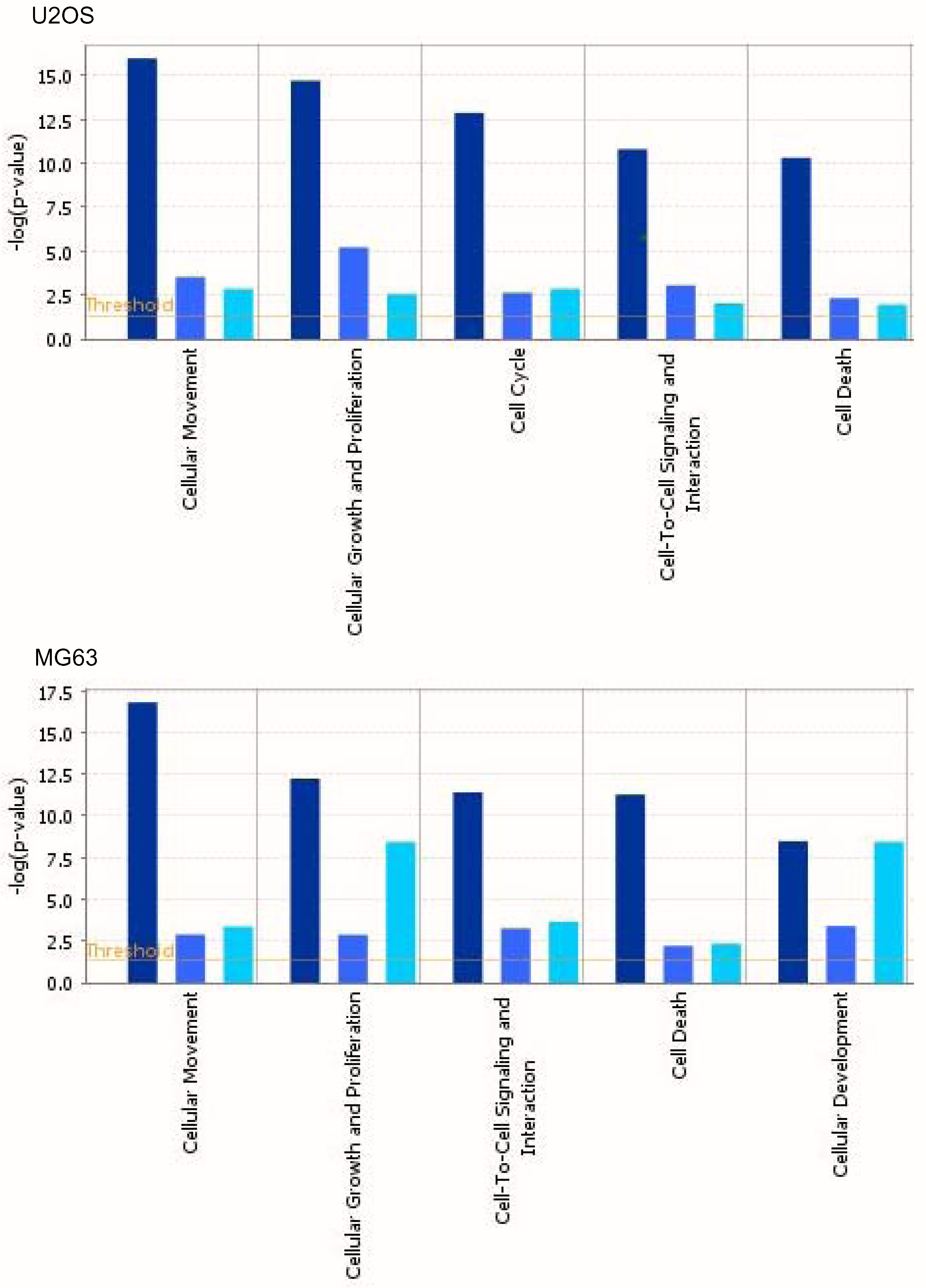

Supplement: Figure S3 — Gene expression, epigenetic, and genetic contribution to cellular function disruption in OS. Top 5 most significantly affected biological functions in relation to the deregulation of gene expression in U2OS and MG63 versus normal osteoblasts were detected using the Ingenuity Pathway Analysis (dark blue bars), and compared to IPA analysis of DNA methylation (light blue bars) and genomic imbalance (turquoise bars) in these cells. The p-value threshold is set at 0.05. (7.98 MB TIF) [file pone.0002834.s003.tif]

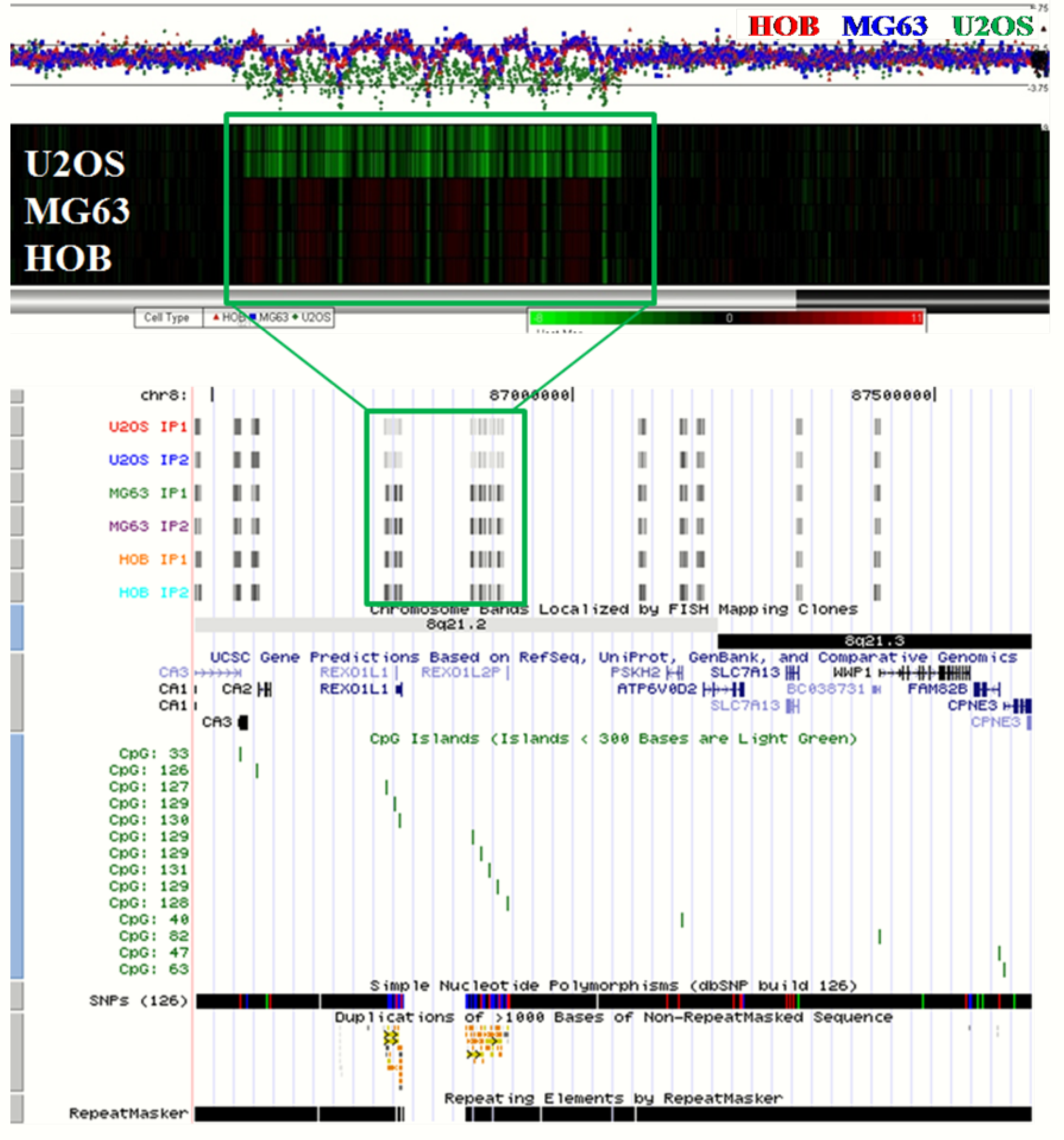

Supplement: Figure S4 — Regional hypomethylation in U2OS cells. Top panel is the PGS-generated region view of the hypomethylated genomic region in U2OS cells located at 8q21.2, featuring the colour-coded profile of the signal from each cell type, and the corresponding heat-map of the replicate array experiments bellow (in log2). Middle panel shows the PGS-generated .wig file of this region imported into UCSC Genome Browser, displaying the corresponding gene, CpG island, and segmental duplication tracks. (10.31 MB TIF) [file pone.0002834.s004.tif]

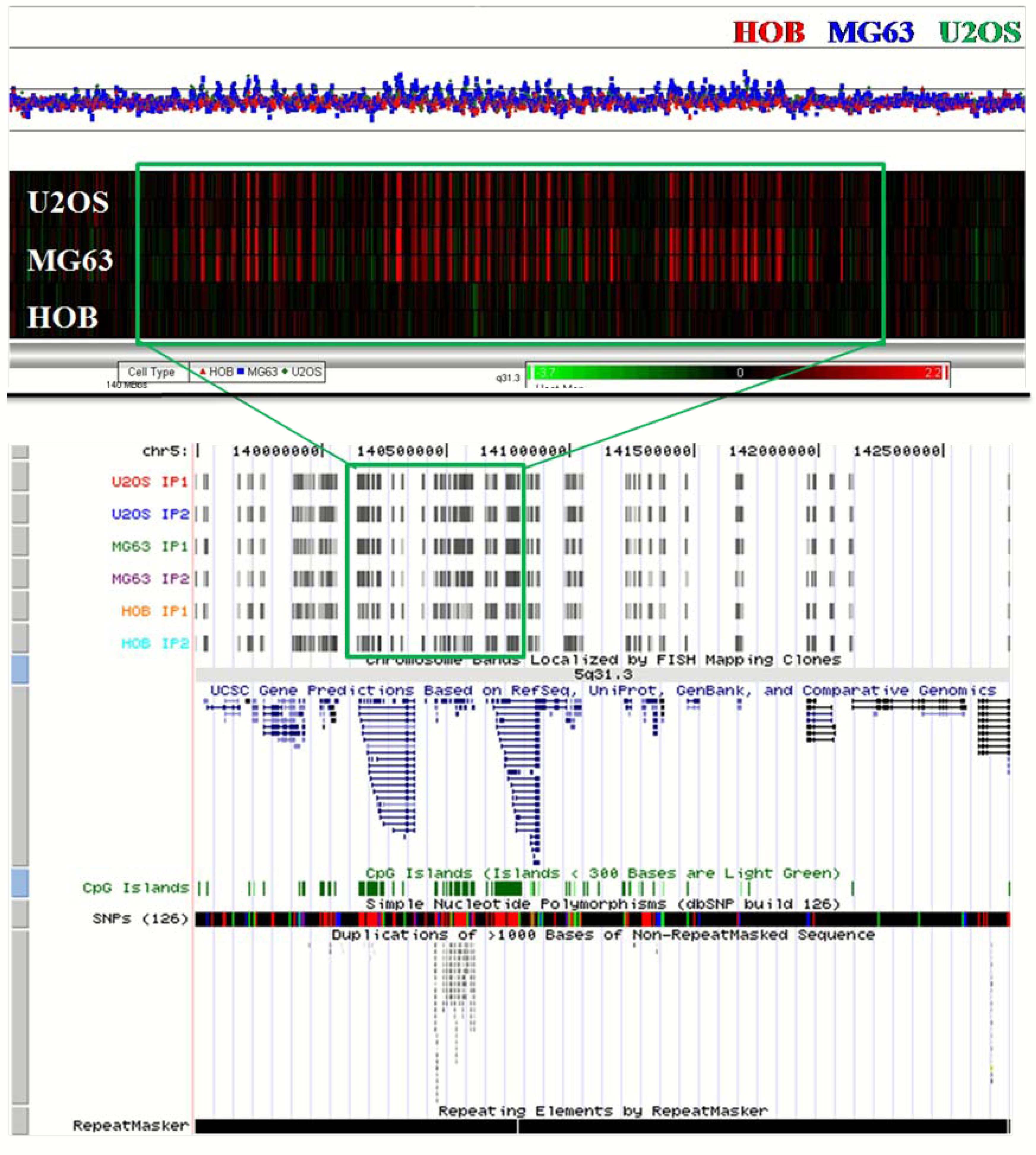

Supplement: Figure S5 — Hypermethylation of Protocadherin gene family in U2OS and MG63 cells. Top panel is the PGS-generated region view of the 2 Mb hypermethylated genomic region in U2OS and MG63 cells located at 5q31.3, featuring the colour-coded profile of the signal from each cell type, and the corresponding heat-map of the replicate array experiments bellow (in log2). Middle panel shows the PGS-generated .wig file of this region imported into UCSC Genome Browser, displaying the corresponding gene, CpG island, and segmental duplication tracks. (15.60 MB TIF) [file pone.0002834.s005.tif]
